# Fabric Vest Socket with Embroidered Electrodes for Control of Myoelectric Prosthesis

**DOI:** 10.3390/s20041196

**Published:** 2020-02-21

**Authors:** Seulah Lee, Babar Jamil, Sunhong Kim, Youngjin Choi

**Affiliations:** Department of Electrical and Electronic Engineering, Hanyang University, Ansan 15588, Korea; seulah@hanyang.ac.kr (S.L.); babar2015@hanyang.ac.kr (B.J.); tjsghd101@naver.com (S.K.)

**Keywords:** textile electrodes, fabric sensor, myoelectric control, embroidered electrodes, posture classification

## Abstract

Myoelectric prostheses assist users to live their daily lives. However, the majority of users are primarily confined to forearm amputees because the surface electromyography (sEMG) that understands the motion intents should be acquired from a residual limb for control of the myoelectric prosthesis. This study proposes a novel fabric vest socket that includes embroidered electrodes suitable for a high-level upper amputee, especially for shoulder disarticulation. The fabric vest socket consists of rigid support and a fabric vest with embroidered electrodes. Several experiments were conducted to verify the practicality of the developed vest socket with embroidered electrodes. The sEMG signals were measured using commercial Ag/AgCl electrodes for a comparison to verify the performance of the embroidered electrodes in terms of signal amplitudes, the skin-electrode impedance, and signal-to-noise ratio (SNR). These results showed that the embroidered electrodes were as effective as the commercial electrodes. Then, posture classification was carried out by able-bodied subjects for the usability of the developed vest socket. The average classification accuracy for each subject reached 97.92%, and for all the subjects it was 93.2%. In other words, the fabric vest socket with the embroidered electrodes could measure sEMG signals with high accuracy. Therefore, it is expected that it can be readily worn by high-level amputees to control their myoelectric prostheses, as well as it is cost effective for fabrication as compared with the traditional socket.

## 1. Introduction

In recent years, there has been a growing interest in myoelectric prostheses, especially, the upper limb, including hand, arm, and shoulder, which is one of the important factors, when doing physical activity. The amputation of the upper limb not only affects physical activity but also emotional or mental issues. In the United States, approximately 3000 people have undergone the loss of an upper limb every year, which has continually increased [1,2]. Amputees in their 20s and 60s occupy approximately 60% of the entire number, and men represent a greater percentage than women [1,3,4]. To meet the needs of upper limb amputees, various prosthetics to assist the amputees’ daily lives have been developed by industries and research institutes. Recently, advances in human–machine interface (HMI) technology have made robotic prostheses available. A myoelectric prosthesis has brought a lot of changes to amputees. It is usually based on surface electromyography (sEMG), which is a method used to obtain the electrical activity of the muscles under the skin surface. The sEMG signal has been actively used when finding out people’s motion intentions.

The state-of-the-art prosthetic hand offers dexterous manipulation to the user, and therefore improves the quality of life. The Touch Bionic Hand, i-Limb Hand, and Michelangelo Hand, as representative robotic hands, are now commercially available. To control these myoelectric prostheses, two methods such as the sEMG approach from the residual limb and the surgical approach have been utilized. First, the sEMG signal approach is the most common method, although the control system based on the sEMG signal is somewhat difficult for implementing dexterous manipulation because the number of electrodes is insufficient. Secondly, a surgical technique, called targeted muscle reinnervation (TMR), has been developed as an alternative. The surgical nerve transfer procedure reinnervates the residual limb nerves into new muscle targets, thus, improving the multiple degrees of freedom of movements of the myoelectric prosthesis [5,6,7]. However, there are a few important restrictions with the surgical approach, for example, people who have a congenital amputation or neurological damage are not able to undergo surgery. Additionally, candidates should have stable soft tissues and an amputation level above the elbow or be within 10 years after the amputation [8]. The aforementioned methods are currently studied to make up for these weak points.

Since the sEMG electrodes are placed inside a prosthetic socket, the fabrication of the prosthetic socket is one of the key elements for prosthetic manipulation and long-term use. The myoelectric prosthesis is dependent on a customized socket for a stably skin–electrode interface. The traditional prosthetic socket consists of rigid support and strap which is designed to wrap around a residual limb. One of the biggest issues of socket production is that the densities of the residual parts such as bone, muscle, fatty tissue, and skin differ depending on the users [9]. The commercially available sockets are known to have several drawbacks. First, they are not suitable for load bearing by fixing a rigid support to the amputated part, and thus dermatological issues often result. Secondly, the prosthetic user feels discomfort, pain, and pressure when the user wears the socket because the traditional shoulder socket should be completely fixed onto the body to help prostheses operate. Third, in the case of younger amputees, their sockets have to be continually customized according to the growth of residual limb since the shape of the residual limb changes over time [10]. Finally, in the case of the amputee who does not have enough residual limb, it is difficult to acquire sEMG signals through the electrodes embedded in the socket. Especially, high-level amputees (such as transhumeral, shoulder disarticulation, and forequarter amputation) need to acquire various sEMG signals because many mechanical joints of the prosthesis should operate including hand, wrist, elbow, and shoulder. For the measurement of the sEMG signal, representative wet and dry types of surface-EMG electrodes are utilized; the wet type is composed of Ag/AgCl as a disposable electrode and the dry type is a metal electrode. The disposable wet electrodes are likely to cause skin irritations and are not reusable due to the adhesive. The dry metal electrodes are usually used as the electrodes for the myoelectric prosthesis. The metal electrodes are embedded inside the socket in the process of making the socket. After the socket is made by a prosthetist, it is difficult to change the shape, size, number, and measurement locations.

With the recent technological advancement, a conductive textile has been rapidly studied with flexible sensors and electrodes. The conductive textile is organized with sensing properties, such as resistive and strain, combined with the traditional textile functions such as lightweight, inexpensive, and flexible. The conductive textile is focused on a variety of fields, especially, it is growing in the biotechnology field for smart healthcare monitoring. The textile sensors as substrate devices are likely to contact with the human body in a noninvasive method, such as shirts [11,12], gloves [13], and band type [14,15]. The textile sensor is classified into four significant areas according to the textile structure and pattern as follows: dyeing [16], knitting [17], weaving [18], and embroidery [19]. In particular, the embroidery technique can make three-dimensional patterns on the textile surface via post processing as compared with the printing, knitting, and weaving techniques. For these reasons, smart fabric is attracting the attention of those interested in secure fastening and sEMG electrode development of the prosthetic socket.

Recently, textile electrodes for the sEMG control of a prosthesis have been gradually studied by researchers. Jiang et al. [14] developed a nonwoven fabric sEMG sensor and conducted the artificial neural network to verify the performance of the developed fabric sensor. Embroidered electrodes have been designed for control of myoelectric prostheses by [20,21]. Most of the proposed electrodes were made either using commercial electrodes or without an adhesive property. In previous studies, we developed the knit band sensor [15], but it was only able to be worn on the forearm. Additionally, commercial sockets such as a fabric shoulder socket (Martin Bionic, Oklahoma city, OK, USA) [22] and the CJ Socket (CJ socket technology, Beverly city, NJ, USA) [23] were manufactured mixing flexible fabric and rigid parts. Although these commercial sockets provided comfort for the amputee, they did not contain the embedded electrodes. 

In this paper, we propose a novel wearable shoulder socket containing an sEMG sensor for high-level upper extremity amputations. The wearable sensor is able to measure sEMG signals using the embroidered electrodes. The proposed electrodes are embedded in vest-type sockets, allowing them to be flexibly attached to the skin. Consequently, we integrated the socket and electrodes as the flexible vest type, which was light, skin-friendly, and free of adhesive.

## 2. Fabric Vest Socket with Embroidered Electrodes 

**Figure 1:**
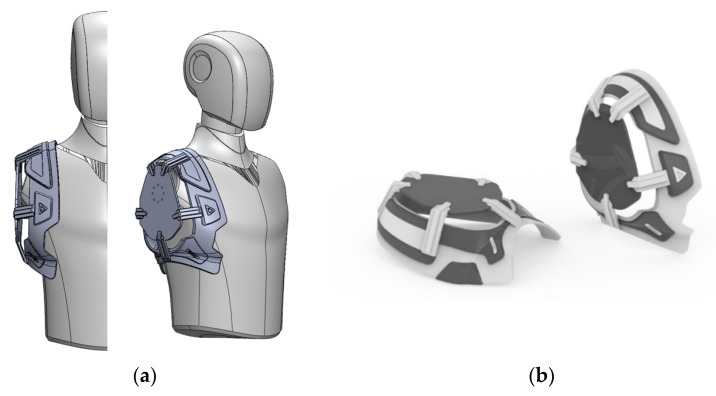
The proposed rigid support of the fabric vest socket. (**a**) three-dimensional (3D) computer-aided design (CAD); and (**b**) rendering design.

For high-level amputees, the fabric vest socket with embroidered electrodes provides a practical alternative to the traditional socket fabricated using only rigid support. The developed socket was fabricated using rigid support and flexible vest which included embroidered electrodes. As shown in Figure 1, the rigid support was made from ABS (acrylonitrile-butadiene-styrene copolymer), which has impact-resistive and tough characteristics. The rigid support was designed using a computer-aided design (CAD) program after scanning the shoulder part of a mannequin, and then it was manufactured by machining ABS material. Since the prosthesis of the high-level amputee is much heavier than that of the low-level amputee, it is essential for the rigid support part to sustain the heavy prosthesis. In addition, the flexible vest was fabricated to disperse its weight on the user’s body, as well as for easy wearability. The traditional socket for shoulder-level prosthetics was composed mainly of rigid parts and strap to fix between the socket and residual limb. However, users suffered pain and pressure in specific areas of the body, as well as it was uncomfortable to wear. In order to solve the issues, the flexible vest was developed using neoprene, which provided cushioning. The neoprene between fabrics was applied using the punching technique for breathability. 

**Figure 2:**
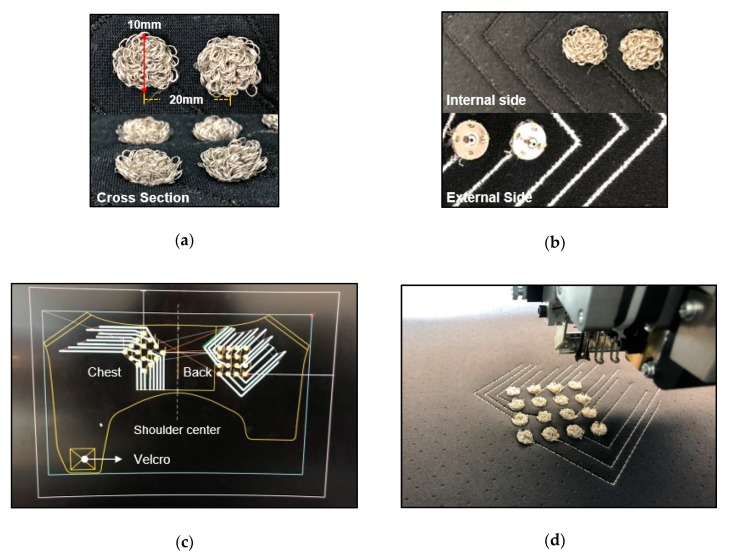
The proposed flexible socket part of fabric vest socket. (**a**) Size of the embroidered textile electrodes; (**b**) internal embroidered textile electrodes and external conductive path; (**c**) two-dimensional (2D) CAD drawing of the flexible socket; and (**d**) fabrication of the embroidered textile electrodes.

The flexible vest includes the conductive yarn of filaments coated with silver. The embroidered electrodes using AMANN Silver-tech 120 conductive yarn have resistance values < 530 ohm/m. The conductive yarn used in the embroidered electrodes was made with silver-coated polyamide and polyester hybrid thread. The multifilament silver-coated yarn is 3-ply with a strength of 111 N/m, additionally, it has antimicrobial characteristics (no cell damaging effect in the cytotoxicity test according to DIN EN ISO 10993-5) [24]. In order to contact skin and electrodes closely, we used the structure of pile embroidery. Pile embroidery as the electrode is the fabric with a three-dimensional soft texture and a loop of yarn on the surface. The electrodes were designed according to the SENIAM (surface EMG for a noninvasive assessment of muscles) and the recommendation suggested by Hermens et al. [25] as a criterion. The embroidered electrode was fabricated as a circle with a diameter of 10 mm, and an interelectrode distance of 20 mm on a bipolar electrode configuration, as shown in Figure 2a. In addition, a conductive path, as the role of an electrical cable, was embroidered back and forth, as shown in Figure 2b. The non-conductive yarn was embroidered on double sides for the electrical insulation, and the conductive yarn was embroidered inside for carrying the sEMG signal. With respect to the flexible vest, the conductive yarn as a textile electrode was embroidered on the neoprene prior to cutting and sewing. As shown in Figure 2c,d, 16-channels (thirty-two electrodes) as textile electrodes were placed on the chest and the back, respectively. These electrodes were located to obtain sEMG signals during the muscle movement of the chest and back considering the shoulder-level amputees. After making the vest socket pattern, we embroidered the electrodes with conductive yarn in the desired positions. 

**Figure 3:**
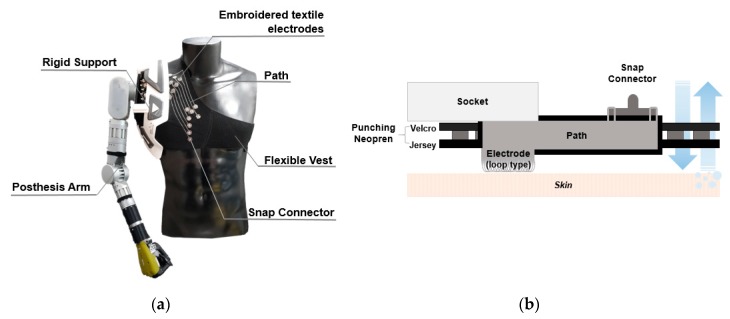
Wearable vest socket with embroidered textile electrodes. (**a**) Prototype of wearable vest socket and prosthetic arm; (**b**) cross-sectional view of wearable vest socket with the embroidered textile electrode.

Then, the wearable vest socket is attached to the rigid support on the flexible vest. In addition, the user can adjust its size using the Velcro. Thus, the textile electrode is able to be used long term without adhesive gel and skin irritation issues [26]. Figure 3 shows a prototype and cross-sectional view of the wearable vest socket. Since the embroidered electrodes are mainly located under the rigid support, it is easily connected with a snap connector through a conductive path. In addition, the punching neoprene provides breathability, lightness, and suppleness.

## 3. Method

For the experiments regarding the electrode performance, a total of 10 able-bodied subjects were recruited in this study. All subjects were healthy with no diseases. Prior to the experiment, the subjects understood the experimental protocol, risk, and benefits of the study. Next, all subjects performed the pretest for 10 min. All methods and procedures were approved by the Institutional Review Board (IRB) on Human Subjects Research and Ethics Committee at the Hanyang University Hospital, Seoul, Korea (July 2019, HYI-16-055-8). 

In order to verify the developed vest socket, three kinds of experiments were conducted. First, the slightly moistened embroidered electrodes (called “embroidered electrodes”) and commercial electrodes were measured on the forearms of all subjects. Second, the skin-electrode impedances of the developed embroidered electrodes, fabric electrodes in our previous research, and commercial electrodes were measured under dry and wet conditions. Finally, the posture recognition experiment was repeatedly demonstrated by five healthy volunteers. All experiments were performed in the same room kept at a constant temperature. Prior to the experiment, the preparation of the skin was done by wiping the measurement location with an alcohol swab. 

To facilitate the sEMG signal processing, an EMG acquisition board including analog amplifiers, three kinds of filters (high-pass, low-pass, and notch filter) and analog-to-digital converters (ADC) was used properly. The sampling time was set to 1000 Hz using INTAN RHS2000 chip for high-speed ADC. For the sEMG performance evaluation, the obtained raw data were filtered with a band-pass filter with cut-off frequencies from 8 to 200 Hz, and a notch filter was used to reduce the 60 Hz power-line interference. For posture classification, the obtained raw data were directly used for the practicality verification of the fabric vest socket without any filtering processes.

The sEMG signals acquired from the developed and the commercial electrodes were analyzed in terms of the signal amplitudes, SNR, and the skin-electrode impedance. In addition, posture recognition was conducted for controlling the myoelectric prosthesis. The experimental analysis was performed using MATLAB R2018a (Mathworks, Natick, MA, USA), SPSS version 21 (IBM SPSS statistics 21, SPSS Inc., Chicago, IL, USA), Python and Pytorch.

### 3.1. sEMG Performance Evaluation 

For the sEMG signal processing, root-mean-square (RMS) operations were sequentially performed with 256 sample windows at 1000 Hz sampling frequency. The root-mean-square (RMS) value was calculated to validate the usefulness of the sEMG signals. The quality of the developed embroidered electrode with that of the commercial electrode was compared with each other. For this purpose, both types of electrodes were placed on the same forearm and the reference electrode was placed on the wrist. According to the researcher’s cue, the subjects were repeatedly requested to perform a relaxation and a grasping motion as MVC (maximum voluntary contraction) for 10 s, respectively. In addition, a 2.25 kg handgrip was used to take exact postures. 

The SNR represents the ratio of signal power to noise power. An SNR is calculated as following from:

(1) $$SNR_{dB}\;=\;20\cdot \log\frac{E_{S}}{E_{N}}$$

where $E_{S}$ represents the average RMS of sEMG signals during the muscle contraction and $E_{N}$ implies the average RMS of sEMG signal at the muscle relaxation. A correlation analysis and a paired sample *t*-test for SNR value between both electrodes were also employed.

### 3.2. Skin-Electrode Impedance Measurement 

The impedance of an electrode is directly in relation to the SNR and performance; thus, it means the electrodes, when in use, need to go through an impedance validation that supports efficient performance. Due to this reason, commercial electrodes are set to play a benchmark role to develop new kinds of electrodes. In consideration of this aspect, we compared the impedance of the embroidered electrodes with a commercial electrode set as the reference benchmark for all the other electrodes. The impedance between the electrodes through the skin was the most important parameter for the signal quality of measured sEMG signals. In order to validate the skin-electrode impedance of embroidered textile electrodes, we measured electric resistance between the electrodes using a commercial LCR meter (GW INSTEK-6020, Taipei, Taiwan), which has the ability to provide real-time measurements of actual resistance between the electrodes. The reference was important because the performance of commercial electrodes is considered optimal; however, these electrodes cannot be integrated into the design level this socket is aimed at. Several procedures were conducted before the actual experiment. First, we calibrated the LCR meter to compensate for its contact wire impedances, as well as other parameters. The sampling of the impedance signal was done at 10 kHz with the excitation voltage of 2 V peak-to-peak. The reason for sampling the conductive fabric is that the sampling frequency to acquire the sEMG signal from most electrodes is over 1 kHz for real-time processing of sEMG data, although there have been many other studies conducted with a sampling frequency of 10 Hz or 20 Hz [19]. To evaluate the performance of the embroidered electrodes, we compared the impedance of the embroidered electrodes with that of the commercial electrodes. The reason for this study is to confirm the impedance difference of electrodes, as well as the time depreciation of electrode impedance. Additionally, we conducted the electrode impedance measurement experiments under moisture conditions, which was expected to happen while in use or to improve performance artificially if required.

**Figure 4:**
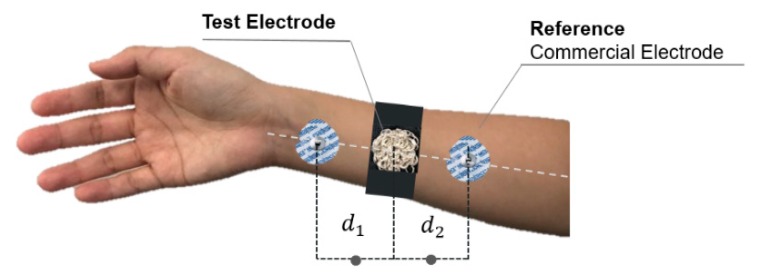
Electrode placement for skin-electrode impedance measurement, where $d_{1}$ and $d_{2}$ are considered as equal distance between the embroidered electrode and the commercial electrodes.

**Figure 5:**
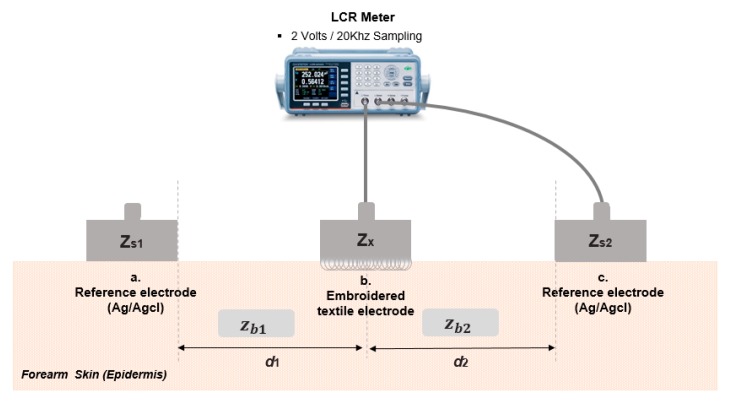
Experimental setup for the skin-electrode impedance measurement.

There are various methods for comparing the impedance measurement of textile and commercial electrodes. Figure 4 shows a reference-based impedance measurement where two reference electrodes are equidistant from the center point used for the electrodes with several conditions. The experiment was considered for various conditions, including dry and wet conditions, as well as knit fabric electrodes from the previous study to compare the performance of each electrode. With regards to previous studies [27,28,29], additional testing on the skin and textile electrode using the equivalent circuit model was conducted. The experimental setup for the skin–electrode interface was used, as shown in Figure 5. An LCR meter was connected to the test and reference electrodes, respectively. The reference electrode was used as the commercial Ag/AgCl electrode (Kendall-MT 100) [30]. Figure 5 shows a general impedance model during the impedance measurement as expected to happen during the EMG signal measurement, where $Z_{s1}$ and $Z_{s2}$ denote the impedances of commercial Ag/AgCl electrodes (reference), $Z_{x}$ expresses the impedance of the developed electrode under specific conditions, and $Z_{b1}$ and $Z_{b2}$ are the skin impedance at the distances *d_1_* and *d_2_*. As mentioned before, *d_1_* = *d_2_*. The overall mathematical model shown in Figure 5 can be described with following mathematical equations:

(2) $$\begin{array}{l} Z_{ab}=Z_{b1}+Z_{s1}+Z_{x}\\ Z_{bc}=Z_{b2}+Z_{s2}+Z_{x}\\ Z_{ac}=Z_{b1}+Z_{b2}+Z_{s1}+Z_{s2} \end{array}$$

It is noted that the developed electrode ($Z_{x}$) is located at the center of reference electrodes. Since we are assuming two electrodes as a reference at the same distance, the set of Equations (2) can be rearranged into the following form giving us the impedance value of the developed electrode.

(3) $$Z_{x}\;=\;\frac{Z_{ab}+Z_{bc}-Z_{ac}}{2}$$

In our experience, $Z_{ab}$ was very similar to $Z_{bc}$ in fact, their difference level remained within a 1% error, and thus we used the following reduced equation in the actual experiment.

(4) $$Z_{x}\;=\;Z_{ab}-\frac{Z_{ac}}{2}$$

### 3.3. Posture Classification for Control Prosthesis 

**Figure 6:**
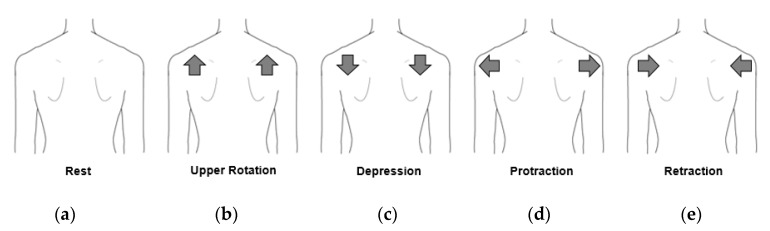
The five different shoulder postures used in the experiments. (**a**) Rest; (**b**) elevation; (**c**) depression; (**d**) protraction; and (**e**) retraction.

The posture classification for controlling the myoelectric prosthesis has been studied by many researchers. It is generally classified by using the user movement intention from the obtained multichannel sEMG signals based on machine learning, artificial neural network, support vector machine, and convolutional neural network. In this study, our posture classification was performed by a convolutional neural network (CNN) with RMS values of sEMG signals as the inputs. It is the most commonly used for sEMG classification [31,32]. The CNN was used to classify postures with sEMG signals extracted from the chest and back. To verify the practicability of the developed fabric vest with the embroidered electrodes, the experiment was repeatedly performed five times for five subjects. The sEMG data were captured from five different shoulder postures such as rest, elevation (upper rotation), depression, protraction, and retraction postures in standing, as shown in Figure 6.

**Figure 7:**
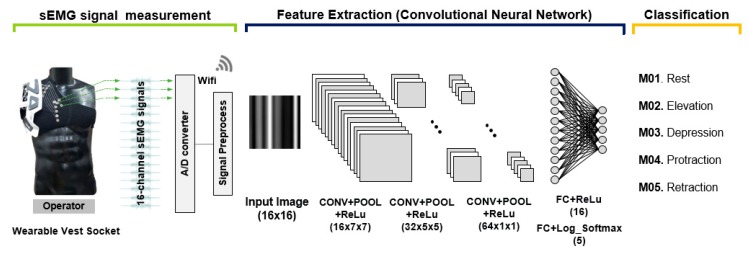
Overview of the proposed shoulder posture classification process.

Figure 7 shows an overview of the proposed shoulder posture classification processes, which consists of three parts, i.e., sEMG signal measurement, feature extraction, and classification. All postures were carried out continuously for ten seconds per posture, having a rest interval between postures of approximately 3 s. The sEMG data were measured at 1 kHz sampling frequency, which underwent smoothing process by using 256 ms moving window RMS. The CNN model for classification was then applied. The sixteen-channel sEMG data obtained by wearing the fabric vest socket were sequentially captured for sixteen samples and each sample was normalized to create 16 x 16 (normalized samples x channels) input images. Thus, the number of total images was approximately 360,000 images for each subject. The data were divided into the training dataset at 75% of the total images and the validation set at 25%. The proposed CNN network consisted of three convolutional layers (CONV), three max-pooling 261 (POOL), and three CNN, two of which were followed by a fully connected layer (FC) with a 262 log softmax functions. While training, 0.5 probability dropout was used to avoid overfitting. After several trials of training, the parameters were set; 10 batch size and 50 epochs. Additionally, a repeated-measures analysis of variance (RM-ANOVA) with Bonferroni adjustment was used to verify whether the five postures were significantly different at the 0.001 level for the 16-channel electrodes. The Greenhouse–Geisser correction for sphericity was adopted.

## 4. Experimental Results 

### 4.1. sEMG Performance Evaluation 

**Figure 8:**
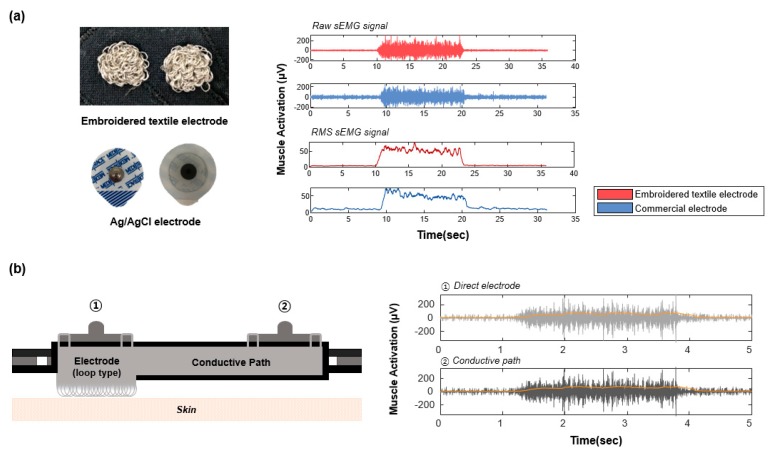
Representative surface electromyography (sEMG) signals. (**a**) sEMG signals obtained from the embroidered textile electrode and the commercial electrode, respectively; (**b**) sEMG signal of the embroidered textile electrode via the direct electrode and conductive path.

The sEMG signals of the embroidered electrodes were analyzed, and the signals were then compared with signals acquired from the commercial electrodes. The representative sEMG signal is illustrated in Figure 8a. Although the sEMG signals of both electrodes indicate a similar amplitude during the muscular contraction, the raw and RMS baseline noise of the developed electrode are more stable than those of the commercial electrode. An additional experiment was conducted to confirm the effect of the conductive path inside the fabric vest socket. The simultaneous measurement of the sEMG signal was conducted (1) via the direct electrode and (2) via the conductive path from the electrode. Since these results suggested that there was little difference between them, we could confirm the EMG signals were unaffected by the conductive path, as shown in Figure 8b.

**Figure 9:**
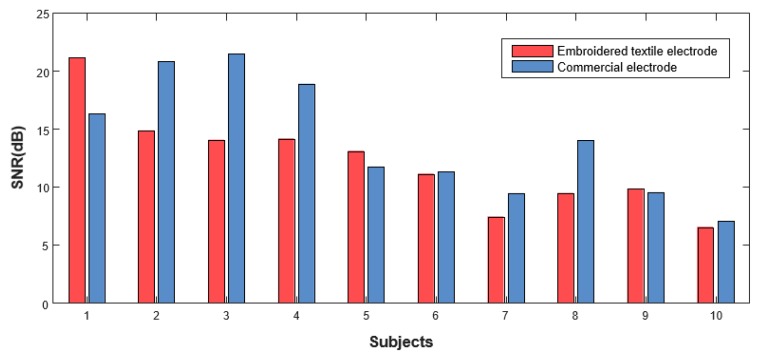
Comparison of the signal-to-noise ratio (SNR) values of the ten subjects.

In order to evaluate the quality of the sEMG signal, the SNR values of the developed electrodes were compared with those of the commercial electrodes. Figure 9 shows the SNR values of the embroidered textile and commercial electrodes for all subjects. The SNR values of both types were approximately from 8 dB to 20 dB. The SNR value of commercial electrodes for most subjects was slightly higher than those developed. Of all subjects, the SNR values of the three subjects presented higher than those of the commercial electrodes. 

As listed in Table 1, the statistical measures of SNR values for both electrodes were derived, including the mean and standard deviation. For the SNR of all subjects, the mean values of the embroidered and commercial electrodes are 12.16 dB and 14.07 dB, respectively, and their standard deviations are 4.27 dB and 5.09 dB, respectively. Among all subjects, on the one hand, the overall SNR values of developed and commercial electrodes are the highest at 21.13 dB and 20.81 dB, respectively. The lowest SNR values, on the other hand, are 6.53 dB and 7.06 dB, respectively. The interrelationship between the SNR values and the two type electrodes variables were analyzed by calculating Pearson’s correlation coefficient. The moderate correlation was found between the SNR of embroidered textile and that of commercial electrodes (R = 0.688; *p* < 0.05). Additionally, there was no statistically significant difference in the mean between the SNR value of both electrodes (*t* = 1.606, *p* = 0.143).

### 4.2. Skin-Electrode Impedance Measurement

**Figure 10:**
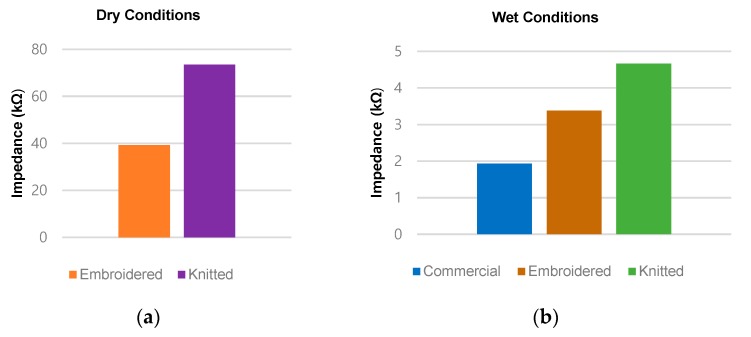
Experimental results for the skin-electrode impedance measurement. (**a**) Dry condition; (**b**) wet condition, in which the impedance of the embroidered electrode has 175% of that of the commercial electrode and that of the knitted electrode has 241% of that of the commercial electrode, respectively.

In order to find the best suitable condition for the use of the embroidered electrode, as well as its impedance performance in comparison with the commercial electrode, the skin-electrode impedance measurement was conducted under dry and wet conditions. Figure 10a shows the impedance of the embroidered electrode and the knitted electrode suggested in our previous paper [15]. It is evident that embroidered electrodes show superior performance to that of knitted electrodes in the dry conditions. In detail, the embroidered electrode impedance is approximately 34 kΩ at the dry condition and it is much lower than that of the knit electrode. For the wet conditions, for the knitted and embroidered electrodes, we artificially made both slightly wet. In this case, the embroidered electrodes have an impedance close to the impedance of the commercial electrode using the electrolytic gel, as shown in Figure 10b. The results, in Figure 10b, also show the performance enhancement technique for the embroidered electrodes through the moistening of electrodes.

**Figure 11:**
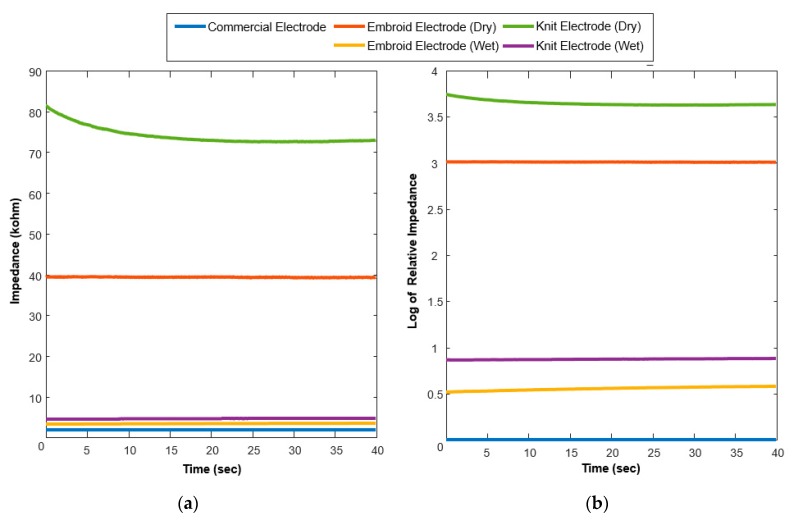
(**a**) Real-time impedance profiles of each electrode such as the commercial electrode, the proposed embroidered electrode in the dry and wet conditions, and the knitted electrode in the dry and wet conditions; (**b**) real-time impedance profiles of each electrode (embroidered electrode in the dry and wet conditions, and the knitted electrode in the dry and wet conditions) relative to the mean impedance of commercial electrode. Where the impedances were calculated after being sampled at 20 kHz frequency.

In order to compare further, Figure 11 shows the real-time impedance profiles according to the time progress for all the electrodes. In the dry conditions, although the impedances of all fabric electrodes are considerably high, it is noted that the impedance of the embroidered electrode is much lower than that of the knitted electrode. Especially, the knitted electrode in the dry condition showed the time-varying property in the early part. For ease of understanding, Figure 11b shows log scales of the relative impedances of all the understudy electrodes. For this purpose, we first calculate the mean impedance of the commercially available electrode over 1 min and denoted as ${\bar{Z}}_{ref}$. In addition, to compare the impedance, a log scale is adopted to magnify the difference between each electrode in comparison with the reference electrode. In other words, let us denote $Z_{elec}^{R}$ as the relative impedance, and $Z_{x}$ as the target electrode impedance, then, we can write the log scales of the relative impedances as follow:

(5) $$Z_{elec}^{R}=\log\left(\frac{Z_{x}}{{\bar{Z}}_{ref}}\;\right)$$

### 4.3. Posture Classification 

To study the practicality aspects of the developed fabric vest socket, we demonstrated by wearing a shoulder socket with embroidery electrodes for controlling myoelectric prosthesis. The CNN model was set for each subject’s own shoulder posture classification, and it was trained and validated using only the subject’s own data, in other words, each subject has their own CNN model. Table 2 shows the average classification accuracy with experiments repeated five times for each subject. The mean accuracy of the posture classification of five subjects was 97.17%. Of the five postures, the depression posture reached 97.60% with the highest classification excluding the rest posture, whereas the upper rotation (elevation) posture showed the lowest accuracy at 95.84%. All postures were over approximately 95%. 

**Figure 12:**
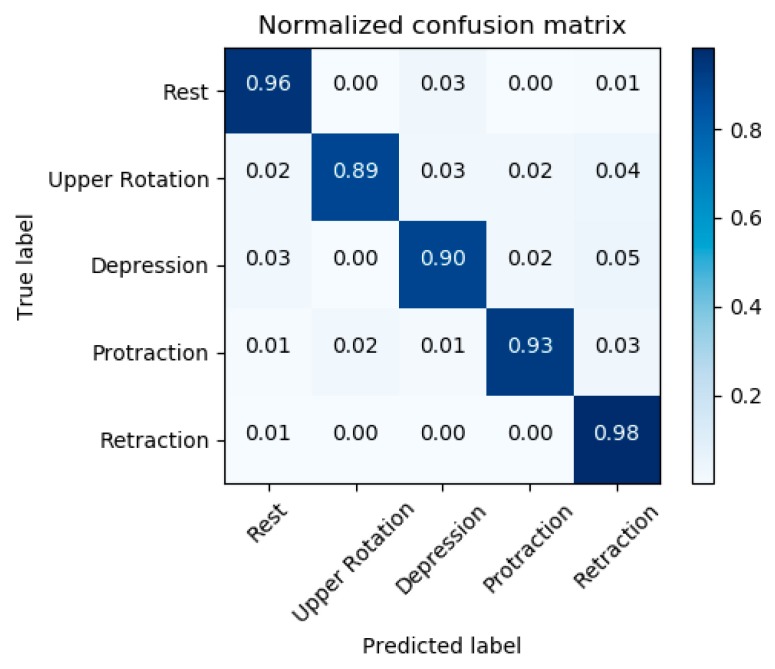
Confusion matrix of five postures where the CNN was trained and verified using all the data collected from five subjects.

**Figure 13:**
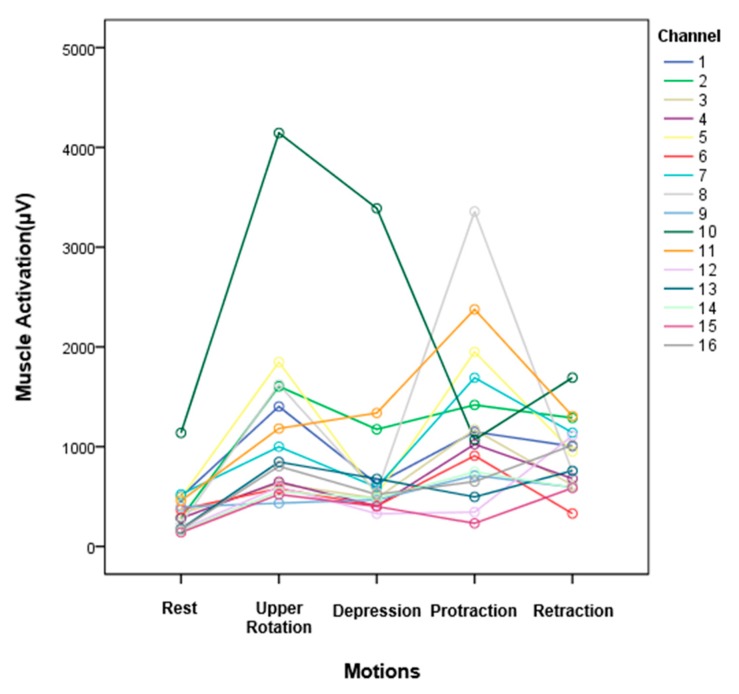
Average root-mean-square (RMS) values of 16-channel sEMG signals of five postures measured from the fabric vest socket.

To further confirm the generalization performance, the new CNN model was trained and verified using all the data collected from all subjects. The total number of data was 1,800,000. Figure 12 shows the confusion matrix regarding five postures of all subjects. The mean accuracy reached 93.2%. The accuracy of all postures was above 90%, except for the upper rotation (elevation). The highest accuracy in all postures was retraction posture at 98%. The upper rotation (elevation) was the lowest accuracy at 89%. In addition, we could confirm the differences in sEMG signals according to five postures, as shown in Figure 13, where 16-channel sEMG signals for five postures were analyzed by RM-ANOVA. Consequently, there were significant differences in five postures between the RMS values of sEMG signals (*p* < 0.001).

## 5. Discussions

In this study, we proposed a fabric vest socket with embroidered electrodes for the myoelectric control of the robotic prosthesis. The traditional socket consists of rigid support such as metal and plastic, which causes not only pain and pressure but is also uncomfortable. In addition, it is still lacking factors such as lightweight, low cost, and multifunctionality. However, the developed socket is easy to wear and lightweight, since it is composed of flexible vest as fabric and rigid support as ABS material. It is also easily worn because of the vest-type design. Users must constantly customize their socket for the changing residual limb over time because the traditional socket is not able to modify after it is made. On the contrary, since the proposed vest socket is made of fabric, it is possible to make some modifications without the help of a prosthetist. In addition, the electrode using the conductive yarn can be embroidered into the desired position, as well as post processing is possible even after the socket is made. The silver-coated yarn used for the electrode is known as an antibiotic material, as well as it has no side effects such as skin trouble and allergy [33]. 

In order to verify the effectiveness of the fabric vest socket, we conducted some of the experiments. First, as shown in Figure 8, the signal amplitude of the developed electrode was observed to be almost similar to that of the commercial electrode. Additionally, the sEMG signal of the embroidered electrodes showed it does not influence a conductive path. Thus, when measuring the signals from wide parts such as chest and back on the body, the conductive path might be efficient because the connectors are able to gather at a particular place. 

Secondly, although the SNR values of the developed electrodes appeared to be only a little lower than that of commercial electrodes, the SNR values of both are within an acceptable range [34]. The SNR value differs between individuals, which could be caused by the difference of individual grasping powers and individual muscular fatigue. It can be seen that the mean SNR value of the commercial electrode (14.07 dB) was higher than that of the embroidered electrode (12.16 dB), on the contrary, the standard deviation of the embroidered electrode (4.27 dB) was higher than that of the commercial electrode (5.09 dB). Therefore, there were no differences in the SNR mean values between embroidered and commercial electrodes (*t* = 1.606 and *p* = 0.143). Both electrodes have similar performance, which could serve as a substitute for the conventional metal electrode. Considering that the commercial electrode is disposable and gel type, the developed electrode can be reusable in practical terms, and thus is remarkable. 

Thirdly, the skin-electrode impedance measurement was carried out in three different conditions to find the best suitable scenario for the use of fabric electrodes, as well as its impedance performance in comparison with the commercial electrodes. In this case, we can, of course, expect the performance of the commercial electrode as a benchmark for performance to be evaluated. However, the embroidered electrodes in slight moisture conditions appeared as effective as the commercial electrodes. This result shows the huge potential for using these kinds of sockets in the future. In this study, it is understood that artificial moisture for these electrodes is not a hardship and, additionally, the moisture in the electrodes does not significantly deteriorate the performance of the sensor. The use of these electrodes can be supported for further study, since these electrodes have superior reusability, comfort, and connectivity, and in addition complex designs and formations can be done using these electrodes. According to changes in impedance over time, we clearly found that there was no significant change in the impedance of embroidered electrodes in the span of observations, however, the impedance of knitted electrode in dry conditions simply made it harder to be used, since its impedance varied with time, as well as disturbance applied externally. In addition, the settling time is considered important in the case of electrodes where the signals are dependent on impedance values.

After validating the embroidered electrodes performance through a comparison with commercial electrodes, we further demonstrated the applicability of the proposed electrodes for the shoulder posture recognition. For the use of sEMG signals, the application including a myoelectric prosthesis requires clear patterns according to postures. The classification using the embroidered electrodes for five subjects was at a high level. Since the classification using data obtained from all subjects reached a high accuracy of over mostly 90%, the generalization performance could be acceptable. 

We should also note that the effects of the socket with embroidered electrodes, in our study, are fundamentally different from those of other fiber electrodes previously considered. Although fabric-based electrode development has been reported previously, it has mostly relied on experiments limited to electrode evaluation. These methods tend to have limited practicality and are not enough to be adapted to the use of various applications. The previous embroidered electrodes [19,20,21,35] have a flat-type design, whereas the developed electrodes stick more closely to the skin as a three-dimensional loop-type design. The shoulder socket embedding the proposed electrodes not only overcomes these issues but also provides a technology that can be easily applied to the HMI, as well as the prosthetic industry. An advantage of our study is that the slightly moisten electrodes performed admirably as compared with the dry condition. Although the slightly moisten electrodes were utilized during the experiment, there were no side-effects, unlike the disposable and metal electrodes.

## 6. Conclusions

This paper presents the fabric vest socket including the embroidered electrodes for myoelectric prosthesis control of shoulder disarticulation. The developed vest socket carries out design for fabrication, EMG signal performance, skin-electrode impedance, and posture classification. We conclude that combining flexible fabric with conductive embroidered electrodes and rigid material is the most effective method for the comfortability of shoulder disarticulation amputees. Unlike a commercial (Ag/AgCl) electrode, the developed electrode is reusable because of the conductive textile. This study is a meaningful attempt to apply practical use on high-level amputated users, as well as targeted muscle reinnervation (TMR), not only were the electrodes inserted in the flexible vest, but the performance was also demonstrated against the disposable electrode. Since the developed electrodes have conductivity, flexible, three-dimensional structure, and are harmless to humans, they can be applied to wearable devices related to electrocardiography (ECG), electroencephalography (EEG), and respiration using information and communications technologies (ICT). The developed vest with embroidered electrodes can also be available in various applications, such as HMI devices, smart wear, etc.

## Figures and Tables

**Table 1:** Total SNR of the ten subjects.

Electrode Type		Subject										Mean	S.D.
		01	02	03	04	05	06	07	08	09	10		
Embro-idered Textile	Signal (μV)	29.67	34.46	34.79	14.53	15.57	15.61	9.90	11.75	14.50	18.54	19.93	9.38
	Noise (μV)	2.62	6.43	9.38	2.88	3.48	4.35	4.23	4.04	4.74	8.78	5.09	2.35
	**SNR (dB)**	21.13	14.83	14.04	14.14	13.08	11.12	7.42	9.46	9.83	6.53	12.16	4.27
Commer-cial (Ag/AgCl)	Signal (μV)	21.33	38.91	45.50	24.59	23.41	21.11	19.48	20.30	17.00	19.63	25.13	9.37
	Noise (μV)	3.26	3.56	3.81	2.79	6.10	5.74	6.66	4.06	5.76	8.52	5.03	1.82
	**SNR (dB)**	16.33	20.81	21.51	18.91	11.75	11.33	9.46	14.02	9.54	7.06	14.07	5.09

**Table 2:** Classification accuracy of shoulder postures using the proposed convolutional neural network (CNN) networks (%).

Posture	Subject					Mean
	01	02	03	04	05	
**Rest**	98.00	98.00	98.60	98.20	99.20	98.40
**Upper Rotation (Elevation)**	96.60	94.80	96.00	97.40	94.40	95.84
**Depression**	98.40	96.20	99.00	97.20	97.20	97.60
**Protraction**	97.80	96.00	97.20	97.60	97.00	97.12
**Retraction**	98.00	96.60	97.20	96.60	96.00	96.88
**Mean**	97.76	96.32	97.60	97.40	96.76	97.17
